# Complete Genome Sequence of an Equine Arteritis Virus Strain Isolated from a Lipizzaner Stallion in 2015 in Serbia

**DOI:** 10.1128/MRA.00250-19

**Published:** 2019-09-05

**Authors:** Delphine Gaudaire, Sava Lazić, Diana Lupulović, Tamas Petrovic, Gospava Lazić, Nicolas Berthet, Aymeric Hans

**Affiliations:** a ANSES-Laboratory for Animal Health in Normandy, Physiopathology and Epidemiology of Equine Diseases Unit, Goustranville, France; b Scientific Veterinary Institute Novi Sad, Novi Sad, Serbia; c Centre International de Recherches Médicales de Franceville, Franceville, Gabon; d Centre National de Recherche Scientifique (CNRS) UMR3569, Paris, France; e Unité Environnement et Risques Infectieux, Cellule d’Intervention Biologique d’Urgence, Institut Pasteur, Paris, France; Portland State University

## Abstract

Here, we report the first whole-genome sequence of an equine arteritis virus (EAV) strain, RS1, isolated from the semen of a Lipizzaner stallion held in the Vojvodina region of Serbia.

## ANNOUNCEMENT

Equine arteritis virus (EAV) is an *Equartevirus* from the *Arteriviridae* family, belonging to the Nidovirales order (1). The EAV genome is made of a single-stranded positive RNA molecule of approximately 12,700 bp. EAV is divided into 2 groups, the North American (NA) group and the European group, and the latter can be divided into two subgroups named European subgroup 1 (EU-1) and European subgroup 2 (EU-2). EAV is one of the major viral pathogens of horses, present worldwide, and can be transmitted by respiratory and venereal routes (2–4). Acutely infected animals may develop a wide range of clinical signs, including pyrexia, depression, anorexia, and edema of the scrotum in stallions. The main consequences of EVA outbreaks are financial losses due to abortions of pregnant mares and death of young foals. Following primary EAV infection, up to 70% of the stallions will carry the virus in their reproductive tract, sometimes for years, and will shed the virus in their semen. Those stallions play a major role in maintaining the virus in the horse population as a reservoir.

EAV RS1 was isolated from the semen of a seropositive Lipizzaner stallion collected in 2014 in the Vojvodina region of Serbia (3). For molecular characterization of the EAV strain, semen was collected with an artificial vagina and centrifuged at 1,500 rpm for 10 min. Then, 150 μl of the supernatant was collected for RNA extraction using QIAmp viral RNA (Qiagen) according to the manufacturer’s instructions. The extracted RNA was then retrotranscribed with SuperScript III reverse transcriptase and random hexamer primers (5). The cDNA fragments were ligated and then amplified using the Phi29 DNA polymerase (6). Amplified DNA was fragmented by ultrasonication using Covaris M220 and was used to construct a genomic library with the NEBNext Ultra DNA library prep kit (New England Biolabs) according to the manufacturer’s recommendations. The sequencing was performed using a MiSeq machine to give 150-bp paired-end reads with a Miseq Nano sequencing kit v2 (Illumina). A total of 4.5 million reads were generated. Among those, 25,000 corresponding to viral sequences were obtained based on a “similarity-based” approach using BLASTN and BLASTX with targeted sequences available in sequence data banks (GenBank accession numbers LC000003 and BAQ56330 to BAQ56337 for nucleotide and protein sequences, respectively) as described previously (7). A total of 25,000 viral reads were selected based on >75% identity over 60 bp with reference genomes. Sequences in each read that did not match the reference genomes were trimmed. Only regions of the reads matching the reference viral sequence (GenBank accession number NC_002532) were used for the whole-genome sequence assembly of the 12,639 bp using SPAdes v3.1.0 with default parameters.

**FIG 1:**
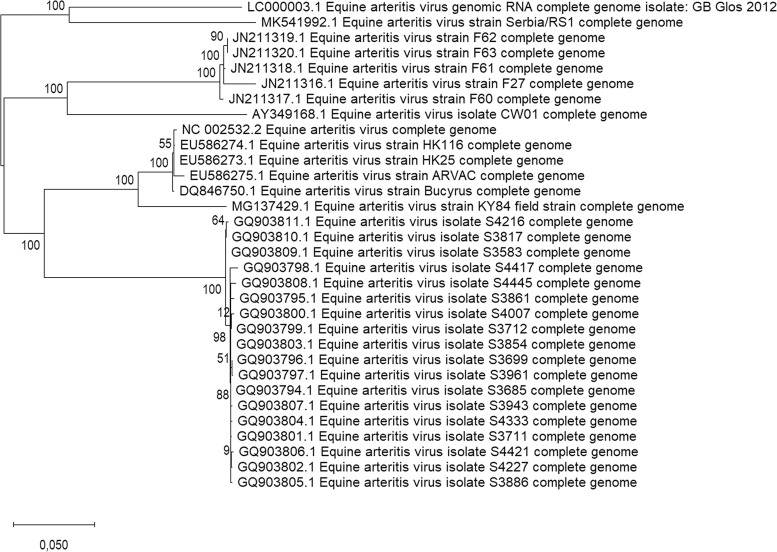
Phylogenetic analysis of the complete EAV genome sequences. The Serbian isolate (GenBank accession number MK541992) clusters with EAV strain GB_Glos_2012 (GenBank accession number LC000003), characterized in the United Kingdom in 2015. A maximum likelihood tree was created using MEGA X. The percentage of trees in which the associated taxa clustered together in the bootstrap test (1,000 replicates) is shown next to the branches. The analysis involved 32 EAV whole-genome sequences retrieved from GenBank.

In conclusion, we report the first whole-genome sequence of a virus coming from the semen of a Lipizzaner stallion held in the Balkan region (more precisely, from Serbia). The evolutionary history was inferred by using the maximum likelihood method based on the Tamura-Nei model using MEGA X software (8, 9), classifying this virus into the European subgroup 2 and forming a new cluster within this subgroup with another EAV isolate, GB_Glos_2012, characterized in 2015 (Fig. 1), which shares 86.2% nucleotide identity (10).

### Data availability.

The nucleotide sequence of the EAV RS1 strain has been deposited in GenBank under the accession number MK541992. The raw sequence reads have been deposited in the NCBI SRA database under the BioProject number PRJNA525421.
